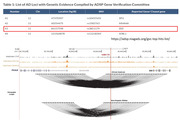# Harnessing chromatin loops to identify susceptibility genes in different populations for Alzheimer’s disease: rs3851179 links to *PICALM* not *EED*

**DOI:** 10.1002/alz.092337

**Published:** 2025-01-03

**Authors:** Liyong Wang, Wanying Xu, Farid Rajabli, Katrina Celis, Leina Lu, Marla Gearing, David A. Bennett, Margaret E Flanagan, Sandra Weintraub, Changiz Geula, Theresa Schuck, William K. Scott, Derek M. Dykxhoorn, Anthony J. Griswold, Margaret A. Pericak‐Vance, Juan I Young, Fulai Jin, Jeffery M. Vance

**Affiliations:** ^1^ John P. Hussman Institute for Human Genomics, Miller School of Medicine, Miami, FL USA; ^2^ Dr. John T. Macdonald Foundation Department of Human Genetics, University of Miami Miller School of Medicine, Miami, FL USA; ^3^ Case Western Reserve University, Cleveland, OH USA; ^4^ Department of Genetics and Genome Sciences, Case Western Reserve University, Cleveland, OH USA; ^5^ Emory University, Atlanta, GA USA; ^6^ Department of Neurological Sciences, Rush Medical College, Chicago, IL USA; ^7^ Northwestern University Feinberg School of Medicine, Chicago, IL USA; ^8^ University of Pennsylvania, Philadelphia, PA USA; ^9^ John P. Hussman Institute for Human Genomics, University of Miami Miller School of Medicine, Miami, FL, USA, Miami, FL USA; ^10^ John P. Hussman Institute for Human Genomics, Miami, FL USA

## Abstract

**Background:**

Annotation of target genes of non‐coding GWAS loci remains a challenge since 1) regulatory elements identified by GWAS can be metabases away from its actual target, 2) one regulatory element can target multiple genes, and 3) multiple regulatory elements can target one gene. AD GWAS in populations with different ancestries have identified different loci, suggesting ancestry‐specific genetic risks. To understand the connection between associated loci (potential regulatory elements) and their target genes, we conducted Hi‐C analysis in frontal cortex of African American (AA) and Non‐Hispanic Whites (NHW) AD patients to map chromatin loops, which often represent enhancer‐promoter (EP) interactions. In our initial analyses, we applied HiC to uncover the regulatory architecture of rs3851179 linked to the embryonic ectoderm development gene (*EED*) in the ADSP gene verification list.

**Method:**

Hi‐C libraries were derived from four AA and four NHW donors matched for age, sex, and *APOE* genotype. The AA samples had 68∼88% African (AF) genome while NHW samples had >95% European (EU) genome. *DeepLoop* was used to generate robust maps of chromatin loops. Hi‐C data from each population were pooled to generate reference chromatin loop maps for the AF and EU genomes.

**Result:**

rs3851179 (OR = 0.9, P = 3.0 × 10^−48^) resides between *EED* and *PICALM*, about 80 Kb away from both with each gene transcribed in opposite directions. Although *PICALM* is designated as a causal gene, *EED* is designated a susceptibility gene by ADSP based on the transcription direction and distance from rs3851179. Hi‐C data from both AF and EU genome in frontal cortex revealed that rs3851179 co‐localizes with a loop anchor, connecting H3K27ac peaks (enhancer) overlapping rs3851179 to the proximal promoter of *PICALM*, suggesting that *PICALM* but not *EED* is the target gene, corroborated by the GTEX e‐QTL data.

**Conclusion:**

This initial application of Hi‐C data demonstrates the utility of chromatin regulatory maps in nominating target genes of GWAS hits in non‐coding loci. The generation and eventual application of Hi‐C maps in African and Amerindian genomes (which are part of the AA or American Hispanic genomic admixture) will be key dissecting ancestry‐specific genetic loci and, thereby, broadening diversity in AD research.